# Transcription Factor *OpWRKY3* Is Involved in the Development and Biosynthesis of Camptothecin and Its Precursors in *Ophiorrhiza pumila* Hairy Roots

**DOI:** 10.3390/ijms20163996

**Published:** 2019-08-16

**Authors:** Can Wang, Chao Wu, Yao Wang, Chenhong Xie, Min Shi, Shivraj Nile, Zhigang Zhou, Guoyin Kai

**Affiliations:** 1 Laboratory of Medicinal Plant Biotechnology, College of pharmacy, Zhejiang Chinese Medical University, Hangzhou 310053, China; 2 College of Fisheries and Life Science, Shanghai Ocean University, Shanghai 200234, China; 3 Institute of Plant Biotechnology, School of Life Sciences, Shanghai Normal University, Shanghai 200234, China

**Keywords:** *Ophiorrhiza pumila*, camptothecin, WRKY transcriptional factor, metabolic regulation, hairy roots

## Abstract

The plant *Ophiorrhiza pumila* produces camptothecin (CPT), a kind of terpene indole alkaloid (TIAs) that has been widely used in treatment of cancer. Tryptophan-arginine-lysine-tyrosine (WRKY) transcription factors have been reported to play important roles in plant metabolism and development. In this study, a novel WRKY transcription factor named *OpWRKY3* was isolated from *O. pumila*, with full-length open reading frame (ORF) of 1128 bp, encoding 375 amino acids. Phylogenetic tree analysis revealed that OpWRKY3 shared the highest homology with VvWRKY30, and it is a significant feature belonging to group III. *OpWRKY3* was responsive to various treatments, including gibberellin (GA_3_), methyl jasmonate (MJ), acetylsalicylic acid (ASA), salicylic acid (SA), and abscisic acid (ABA). Besides, *OpWRKY3* is expressed predominantly in stems. Subcellular localization analysis showed that OpWRKY3 localized in the nucleus. The biomass of *OpWRKY3-SRDX* transgenic hairy roots (S line) was visibly suppressed, while there were slight changes between overexpression of the *OpWRKY3* line (OE line) and the control. In addition, the concentration and total production of camptothecin precursors including loganin and secologanin were significantly changed in both OE and S lines while total production of CPT was significantly changed in most transgenic lines. Thus, the present work revealed that *OpWRKY3* may act as a regulator in the growth and development of *O. pumila*, and in production of camptothecin and its precursors.

## 1. Introduction

Camptothecin (CPT) is a well-known terpene indole alkaloid (TIA), which was isolated for the first time by M. E. Wall and M. C. Wani in 1966 from *Camptotheca acuminata* [1]. CPT has been found in some distantly related plants, including *C. acuminata*, *Ervatamia heyneana*, *Merrilliodendron megacarpum*, *Nothapodytes foetida*, *N. nimmoniana*, and several *Ophiorrhiza* species [2,3,4,5]. CPT exhibits excellent anti-tumor activity by inhibiting the activity of topoisomerase I [1,6]. Its two semisynthetic water-soluble derivatives, irinothecan and topothecan, were approved by the US Food and Drug Administration (FDA) in 1994 and are used extensively for the treatment of ovarian cancer, lung cancer, metastatic colorectal cancer, and cervical cancer throughout the world [7,8,9]. Like many TIAs, CPT is derived from the shikimate pathway and iridoids biosynthesis pathway, which supply the precursor’s tryptamine and secologanin for CPT synthesis [5,10]. The terpene part of CPT is received from the universal precursor’s isopentenyl pyrophosphate (IPP) and dimethylallyl diphosphate (DMAPP), which were derived through the methylerythritol phosphate (MEP) and mevalonate (MVA) pathways. Geranyl pyrophosphate (GPP) was catalyzed by a series of enzymes, including geraniol synthase (GES), geraniol-8-oxidase (G8O), 8-hydroxy-geraniol oxidoreductase (8-HGO), iridoid synthase (IS), iridoid oxidase (IO), 7-deoxyloganetic acid UDP-glucosyltransferase (7-DLGT), 7-deoxyloganic acid hydroxylase (7-DLH), loganic acid *O*-methyltransferase (LAMT), geraniol-10-hydroxylase (G10H), 10-hydroxy-geraniol oxidoreductase (10-HGO), and secologanin synthase (SLS), finally forming secologanin [5,11]. In this pathway, the enzyme nicotinamide adenine dinucleotide phosphate (NADPH): cytochrome P450 reductase (CPR) is essential for the activity of G10H and SLS, which plays a vital role in electron transfer from NADPH to cytochrome P450 [12]. Subsequently, tryptamine and secologanin catalyzes into strictosidine by strictosidine synthase (STR), and strictosidine was converted via strictosidine beta-glucosidase (SGD) into strictosamide [1,5]. The later specific stages of how strictosamide was metabolized to CPT biosynthesis remain unknown [10,13]. At present, camptothecin is mainly extracted from plants; however, the low accumulation of CPT in plants cannot meet the expanding demand of the market [14,15,16]. *O. pumila* is a member of *Rubiaceae*, a medically herbaceous plant that provides an excellent platform for metabolic engineering of CPT biosynthesis [3]. So far, several genes were overexpressed or repressed in the hairy roots of *O. pumila*, confirming the function of the genes in camptothecin biosynthesis. For instance, suppression of *OpTDC* or *OpSLS* resulted in decreased production of CPT and some other metabolites in *O. pumila* hairy roots, revealing that *OpTDC* and *OpSLS* catalyzed the rate-limiting steps in CPT biosynthesis [17]. *STR* or/and *G10H* from *Catharanthus roseus* introduced in *O. pumila* hairy roots led to a significant promotion of CPT compared with the control line [18]. Nevertheless, limited information is available about the regulatory mechanism of camptothecin biosynthesis in *O. pumila*.

As a large family of plant-specific transcription factors, WRKY proteins contain the highly conserved tryptophan-arginine-lysine-tyrosine (WRKY) domain, which is approximately 60 amino acids long with the highly conserved amino acid sequence WRKYGQK at its *N*-terminus and a C2H2 or C2HC zinc finger motif at its C-terminus [19,20]. WRKYs can be separated into three major groups in accordance with the number of WRKY domains and the features of their zinc finger-like motifs [21,22]. Based on the primary amino acid sequence, group II can be further categorized into five subgroups (IIa, IIb, IIc, IId, and IIe) [21,23]. Almost all WRKY proteins can recognize and bind to W-box cis-elements [(T)TGAC(C/T)] in the promoter region of their target genes and can act as transcriptional activators or repressors in regulatory cascades [19,24,25]. Previous studies demonstrated that WRKY transcription factors (TFs) play an important role in multiple physiological activities, including plant growth and development, senescence, biotic and abiotic stress responses, secondary metabolism, and phytohormone signaling [26,27,28,29]. Previous research shown that *AtWRKY75* is a negative regulator of root hair development, the number and length of root hairs showed an increase in its knockout mutant compared with the wild type [30]. Knockout of the *AtWRKY41* mutant significantly downregulated abscisic acid insensitive 3 (ABI3) and influenced the seed dormancy, through binding to three adjacent W-boxes in the promoter of the ABI3 [31]. *Hylocereus polyrhizus WRKY3* (*HpWRKY3*) was associated with sugar accumulation of pitaya fruit by activating the transcriptions of sucrose metabolic genes [32]. In another study, overexpression of *Salvia miltiorrhiza WRKY1* (*SmWRKY1*) increased five-fold the tanshinone production in transgenic lines through binding to the W-box elements of the promoter 1-deoxy-d-xylulose-5-phosphate reductoisomerase (*SmDXR*) involved in the methylerythritol phosphate (MEP) pathway [33]. Overexpression of *SlWRKY45* enhanced tomato susceptibility to the root-knot nematode *Meloidogyne javanica*, which was associated with decreased expression of salicylic acid (SA) and jasmonic acid (JA) marker genes [34]. In addition, *PbrWRKY53* gene of *Pyrus betulaefolia* positively regulated the enhanced tolerance to drought stress by regulating *PbrNCED1* expression [35]. So far, from all these previous studies, it may conclude that WRKY proteins play important roles in multiple physiological activities. However, the functions of some *WRKY* genes, particularly in *O. pumila*, still remain unexplored.

Here, in this study, a novel gene *OpWRKY3* encoding for WRKY transcription factor was isolated from *O. pumila* and functionally characterized. OpWRKY3 shared high homology with VvWRKY30, VqWRKY52, PtWRKY30, AcWRKY41, and AtWRKY30. OpWRKY3 possessed one WRKY domain and a C2HC motif and was classified into group III. The biomass of repression *OpWRKY3* transgenic hairy roots was significantly reduced by suppressing the growth and development of root tip. While there were slight changes observed in *OpWRKY3*-overexpressing lines. In addition, overexpression of *OpWRKY3* in hairy roots resulted in increased concentration and total production (hairy roots and medium) of loganin and decrease of secologanin contentration and production in hairy roots, while repression of *OpWRKY3* resulted in opposite effect compared with the overexpressed lines. The overexpression of *OpWRKY3* resulted in increased the total production of CPT while repression of *OpWRKY3* suppressed significantly the CPT production. Thus, the findings of this study will serve as an accentuating reference for future research on the regulators of alkaloid production in various medicinal plants.

## 2. Results

### 2.1. Bioinformatics and Molecular Characteristics of OpWRKY3

**Figure 1:**
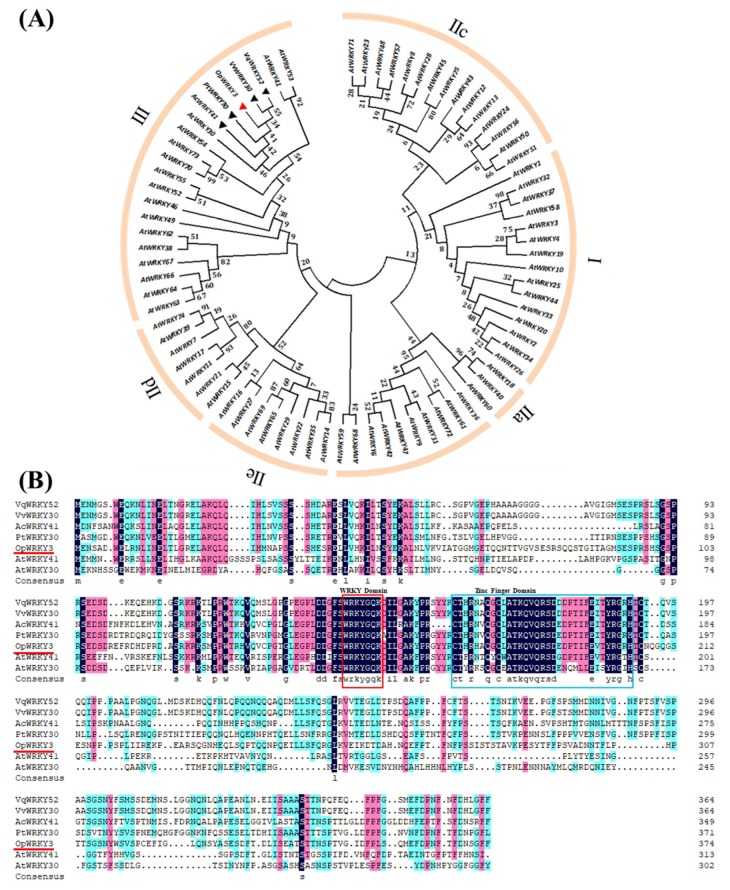
Multiple sequence alignment and phylogenetic analysis of OpWRKY3. (**A**) Phylogenetic analysis of WRKY transcription factors. OpWRKY3 is highlighted with a red triangle. The phylogenetic tree was constructed with the neighbor-joining test using MEGA 7.0. (**B**) Multiple alignment of OpWRKY3 with other WRKY members. The following proteins were used for analysis: VqWRKY52 (AQS95512.1), VvWRKY30 (ALM9663.1), AcWRKY41 (PSS28797.1), PtWRKY30 (AZQ19316.1), AtWRKY41 (AAL35289.1), and AtWRKY30 (NP_568439.1). Identical and similar amino acids are shaded in black and red, respectively. The WRKY domains and the zinc-finger structures are boxed and marked by squares, respectively.

In this study, *OpWRKY3* was cloned and functionally characterized from *O. pumila*. It owned a full-length open reading frame (ORF) of 1128 bp, encoding a protein with 375 amino acid residues, calculated molecular weight of 41.95 kDa, and isoelectric point of 5.77. BLASTp search (NCBI website) revealed that OpWRKY3 shared 55.53%, 54.85%, 53.77%, and 53.51% identity with PtWRKY30, AcWRKY41, VqWRKY52, and VvWRKY30, respectively. Based on the OpWRKY3 and other species’ WRKYs, a phylogenetic tree was constructed and it showed that they could be classified into three major groups, and OpWRKY3, together with AtWRKY30 and AtWRKY41, belongs to group III (Figure 1A). Multiple sequence alignment indicted that OpWRKY3 possesses a highly conserved WRKY domain (WRKYGQK) and a C2H2-type zinc-finger structure (Figure 1B).

### 2.2. Tissue and Induction Expression Profiles of OpWRKY3

**Figure 2:**
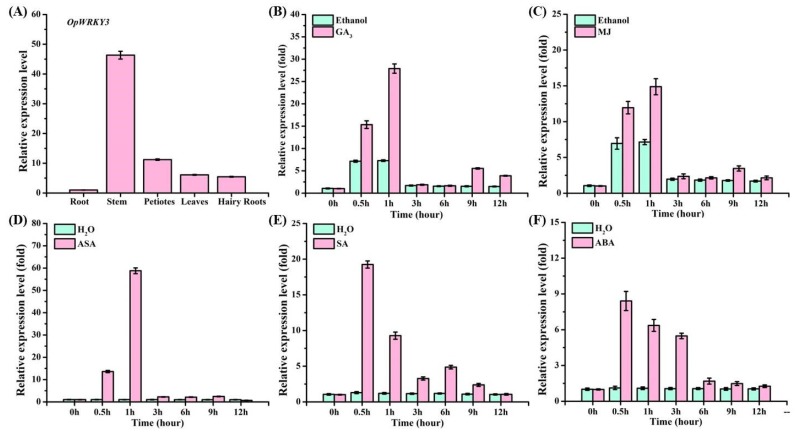
The expression pattern of *OpWRKY3*. (**A**) Expression pattern of *OpWRKY3* in different tissues. Transcript abundance of *OpWRKY3* is normalized to actin by the method of 2^−ΔΔCt^. (**B**) Time course of the expression level of *OpWRKY3* after gibberellin (GA_3_) treatment as determined by qRT-PCR. (**C**) The expression level of *OpWRKY3* after methyl jasmonate (MJ) treatment for selected time points measured by qRT-PCR. (**D**) The expression level of *OpWRKY3* after acetylsalicylic acid (ASA) treatment for selected points by qRT-PCR analysis. (**E**) Time course of the expression level of *OpWRKY3* after salicylic acid (SA) treatment as determined by qRT-PCR. (**F**) The expression level of *OpWRKY3* after abscisic acid (ABA) treatment for selected time points measured by qRT-PCR, respectively. Values are means ± standard deviation of triplicate analyses.

To investigate the tissue expression pattern of *OpWRKY3*, roots, stems, petioles, leaves, and hairy roots were analyzed. Figure 2A reveals that high expression of *OpWRKY3* was found in stems only, and the others have little expression.

To investigate whether *OpWRKY3* responded to exogenous elicitor treatment, 30-day old hairy roots were treated with different inducers, including GA_3_, MJ, ASA, SA, and ABA. The hairy roots were harvested at different time points. The results showed that the exogenous GA_3_, MJ, or ASA significantly induced *OpWRKY3* expression, reaching a peak at 1 h (Figure 2B–D), and thereafter declined rapidly. In addition, the transcript level of *OpWRKY3* was raised rapidly after SA or ABA induction, with a maximum peak at 0.5 h, followed by a gradual decrease (Figure 2E,F). These results indicate that OpWRKY3 can respond to exogenous elicitors.

### 2.3. Subcellular Localization of OpWRKY3

**Figure 3:**
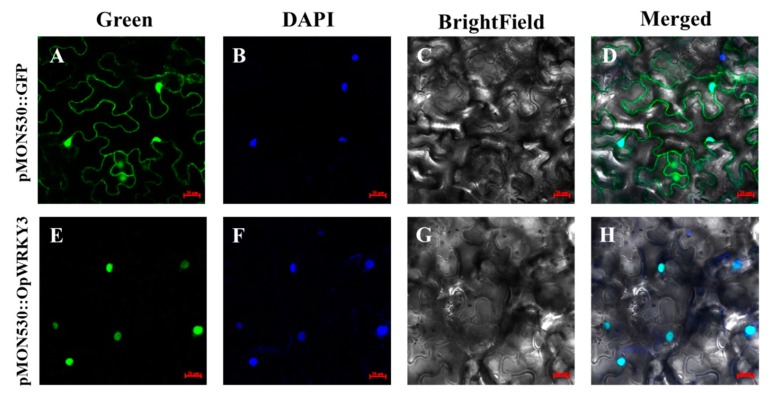
Subcellular localization of OpWRKY3. (**A**–**D**) The free green fluorescent protein (GFP) expressed in tobacco leaves. (**E**–**H**) pMON530:OpWRKY3 expressed in tobacco leaves.

To experimentally confirm the subcellular localization of OpWRKY3, *OpWRKY3* was cloned into the pMON530 vector (the binary vector) and fused with the green fluorescent protein (GFP) reporter gene to generate vector pMON530-OpWRKY3-GFP. Then, the constructed vector and the pMON530 (used as the control) was transformed into the ASE strain and expressed in tobacco leaves, respectively. In the leaves of the transformed control vector plant, the fluorescence of the GFP was detected in the cytoplasm and nucleus, whereas the fluorescence of the OpWRKY3/GFP fusion protein was exclusively found in the nucleus (Figure 3). The expression pattern was consistent with the character of OpWRKY3 as a TF.

### 2.4. Acquisition of OpWRKY3 Transgenic Hairy Roots

To further investigate the function of *OpWRKY3* in *O. pumila*, the *Agrobacterium rhizogenes* strain C58C1 harboring *OpWRKY3* or *OpWRKY3-SRDX* fusion gene were used to infect *O. pumila* explants (Figure S1). The empty vector was used as a control. The positive lines carrying the *OpWRKY3* gene were verified by PCR. The expression of *OpWRKY3* in transformed hairy roots was determined by qRT-PCR. The gene expression levels of *OpWRKY3* in overexpression transgenic hairy root lines were found to be 15 to 360 times higher than in the empty vector control-transformed lines (Figure S2A). Five high-expression lines, including OE-1, 8, 11, 13, and 14, were chosen for further analysis. In *OpWRKY3-SRDX* overexpression lines, *OpWRKY3* was expressed 15 to 110 times higher compared to the empty vector (control) transformed lines (Figure S2B). The five high expression lines, including S-3, 10, 11, 13, and 15, were chosen for further analysis.

### 2.5. Autofluorescent Analysis of Camptothecin in Transgenic Hairy Roots

**Figure 4:**
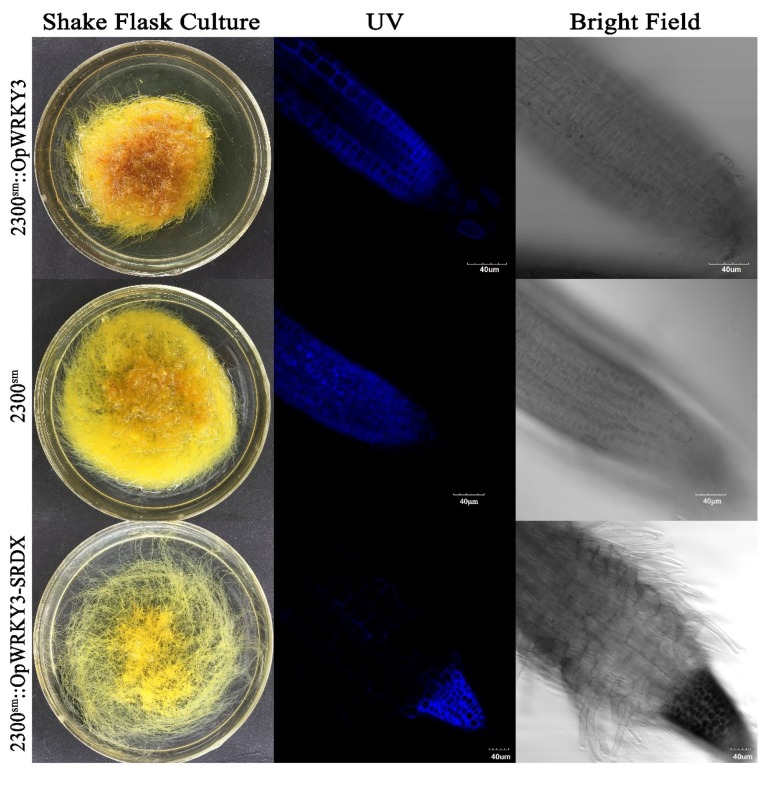
The *OpWRKY3* transgenic hairy roots of camptothecin distribution in the root tip.

Camptothecin can be autofluorescent, which provides a direct way to observe the distribution of CPT in hairy roots. Besides, relative concentrations of CPT in different hairy roots can be examined by comparing the size of the fluorescence area and the intensity of fluorescence. The selected root tips were collected from OE-14, S-10, and EV (transformed empty vector) transgenic hairy roots. The area of fluorescence at the root tip was significantly smaller in *OpWRKY3-SRDX* overexpression hairy roots compared with the control (Figure 4). The root tips of the S-10 line were extremely shortened with a decreasing number of branches in the root-hair zone. However, the fluorescence pattern showed no significant difference between OE-14 and EV hairy root tips. These results indicated that *OpWRKY3* may be involved in the regulation of hairy root growth and root tip morphogenesis.

### 2.6. Metabolism of Camptothecin and Intermediate Compound in Hairy Roots Overexpressing or Repressing OpWRKY3

Based on the expression level of *OpWRKY3*, the biomass of chosen hairy roots lines was analyzed. The fresh weight (1.278 g) and dry weight (0.042 g) were significantly decreased in *OpWRKY3* repression transgenic hairy roots, compared to the control (5.5 g, 0.30g) (Figure S3). The fresh weight (6.148 g) of *OpWRKY3* overexpression transgenic hairy roots was significantly increased whereas dry weight (0.312 g) was not significantly different compared to the control. Among them, dry weight of OE-8, 13, 14 lines was slight increase. The results indicated that repression of *OpWRKY3* inhibited the growth of hairy roots, and *OpWRKY3* may be a transcription factor involved in growth and development of hairy roots. 

To study whether *OpWRKY3* participated in the regulation of camptothecin biosynthesis, the concentration and total production of camptothecin and three intermediate compounds (tryptamine, loganin and secologanin) in *OpWRKY3* transgenic hairy roots and medium were investigated. Compared with OE lines an opposite accumulation pattern was observed for each compound in S lines. The overexpression of *OpWRKY3* resulted in increased significantly lognin concentration in transgenic hairy roots while repression of *OpWRKY3* decreased the lognin concentration, compared to control lines (Figure S4B). In contrast, the secologanin concentration was down-regulated significantly by 70% in OE lines, whereas it was up-regulated significantly by 1.7-fold in S lines (Figure S4C). The tryptamine concentration was slight change in OE lines while it was decreased significantly in S lines (Figure S4A). In addition, the overexpression or repression of *OpWRKY3* showed no significant change in the concentration of camptothecin, compared to the control (Figure S4D). 

**Figure 5:**
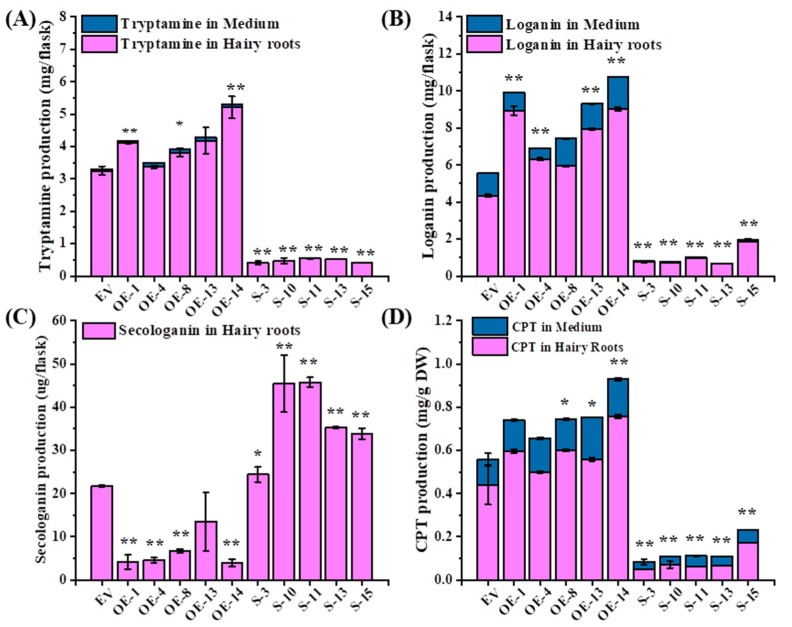
The total production of CPT and its precursors in *OpWRKY3* transgenic hairy roots. (**A**) The total production of tryptamine in OpWRKY3 transgenic hairy roots and medium by high performance liquid chromatography (HPLC); (**B**) The total production of loganin in *OpWRKY3* transgenic hairy roots and medium by HPLC; (**C**) The total production of secologanin in *OpWRKY3* transgenic hairy roots by HPLC; (**D**) The total production of CPT in *OpWRKY3* transgenic hairy roots and medium by HPLC. Values are means ± standard deviation of triplicate analyses. ∗, significant at *P* < 0.05, ∗∗, highly significant at *P* < 0.01.

The total production (hairy roots and medium) of tryptamine, loginin and CPT were up-regulated significantly by 1.28, 1.59, 1.37-fold in OE lines while the total production of tryptamine, loginin and CPT were down-regulated significantly by 86%, 81%, and 77% in S lines, compared to control lines (Figure 5). Among them, the CPT production was increased significantly in line OE-8, 13, and 14, while CPT production was slightly high in line OE-1 and 4 (Figure 5D). However, the overexpression of *OpWRKY3* resulted in decreased significantly secologanin production in transgenic hairy roots while repression of *OpWRKY3* increased significantly the secologanin production (Figure 5C). These results suggested that *OpWRKY3* may be related to the biosynthesis of tryptamine, loganin, secologanin and camptothecin.

### 2.7. Expression of Camptothecin Biosynthetic Genes in OpWRKY3 Transgenic Hairy Roots

**Figure 6:**
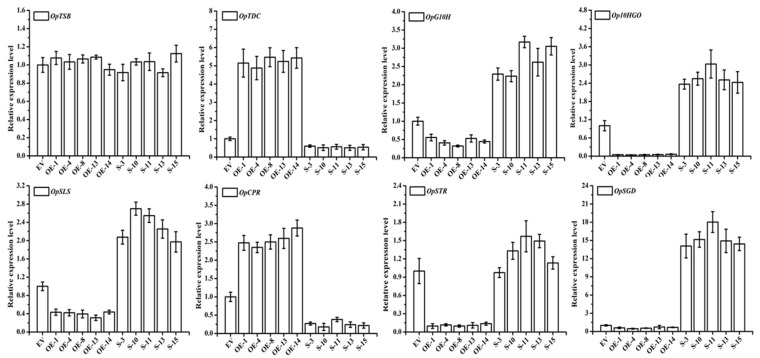
Relative expression levels of selected genes in *OpWRKY3* transgenic hairy roots. Expression levels of these genes were normalized to the empty vector control line. The *actin* gene was used as an internal control. Values are means ± standard deviation of triplicate analyses. *OpTSB*, *OpTDC*, *OpG10H*, *Op10HGO*, *OpSLS*, *OpCPR*, *OpSTR*, and *OpSGD* genes are involved in camptothecin biosynthesis.

To identify the camptothecin biosynthetic genes regulated by *OpWRKY3* in *O. pumila*, transcript levels of eight genes (*OpTSB*, *OpTDC*, *OpG10H*, *Op10HGO*, *OpSLS*, *OpCPR*, *OpSTR* and *OpSGD*) were analyzed by qRT-PCR (Figure S5). Compared with the empty vector (control), the *OpTDC* and *OpCPR* genes were up-regulated in *OpWRKY3* overexpressing hairy roots, which were approximately 5-fold and 2.5-fold more respectively as compared with the control (Figure 6). However, *OpG10H*, *Op10HGO*, *OpSLS*, *OpSTR* and *OpSGD* genes were down-regulated in the five independent *OpWRKY3* overexpression hairy roots. In contrast, *OpTDC* and *OpCPR* genes were significantly decreased in the five independent *OpWRKY3-SRDX* lines, while *OpG10H*, *Op10HGO, OpSLS*, *OpSTR* and *OpSGD* genes were significantly increased compared with control. In addition, the *OpTSB* gene expression level of transgenic hairy roots was no significant change. All these results suggested that *OpWRKY3* may be involved in the regulation of camptothecin biosynthesis.

### 2.8. Activation of the Expression of OpCPR by OpWRKY3

**Figure 7:**
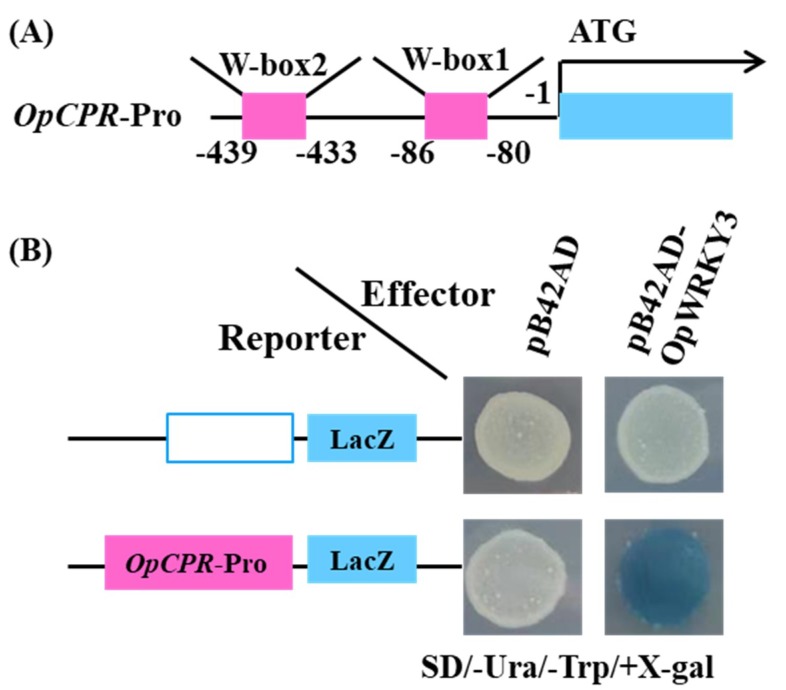
OpWRKY3 binds to the W-box of the *OpCPR* promoter and activates *OpCPR* expression. (**A**) The analysis of motifs in *OpCPR* promoter. (**B**) Yeast one-hybrid assay showed that OpWRKY3 binds to the W-box of *OpCPR* promoter.

Expression profiles showed that the overexpression of *OpWRKY3* resulted in increased the expression of *OpTDC* and *OpCPR*. By analyzing the promoter sequence of *OpTDC* and *OpCPR* genes, we found that there was no W-box on the *OpTDC* promoter and *OpCPR* promoter contained two W-boxes (W-box 1, 2). The W-box 1 and W-box 2 of *OpCPR* promoter were located between positions -80 and -86, -433 and 439, respectively (Figure 7A). Y1H assay showed that OpWRKY3 was bound to the W-box 1 of *OpCPR* promoter (Figure 7B). These results indicated that OpWRKY3 increased the transcription of *OpCPR* by binding to the W-box 1 of its promoter.

## 3. Discussion

In *O. pumila*, WRKY3 transcription factor shared high identity with AtWRKY30, AtWRKY41, and VvWRKY30. Furthermore, OpWRKY3 possesses a highly conserved WRKYGQK motif and a C2H2-type zinc-finger structure, belonging to the group III category. The group III WRKYs, such as *Arabidopsis thaliana WRKY75* and *WRKY41*, have been shown previously and played essential roles in regulating plant growth and development [30,31]. In addition, knockout of the *AtWRKY75* mutant increased the number and length of root hairs [30]. *AtWRKY46*, *AtWRKY54*, and *AtWRKY70* are involved in both brassinosteroid-regulated plant growth and drought response [36,37]. In the present study, the results of *OpWRKY3* overexpression and repression revealed that the expression level of *OpWRKY3* was linear with the biomass of transgenic hairy roots. Repression of *OpWRKY3* inhibited the growth and development of the root tip and ultimately decreased the biomass of transgenic hairy roots. This is consistent with the *WRKY* transcription factor gene *CbNN1* increased rooting ability, promoting adventitious root formation [38]. These results indicated that *OpWRKY3* is involved in the growth and development of hairy roots. In addition, previous reports shown that group III WRKYs possessed the function of regulating secondary metabolism [39,40]. In another study has been shown that overexpression of *CrWRKY1-SRDX* decrease cellular tryptophan decarboxylase (TDC) activity and tryptamine accumulation in transgenic hairy roots [40]. However, their characteristics and functions have not been identified in *O. pumila*. Therefore, the function of WRKYs in *O. pumila* requires in-depth research, especially *OpWRKY3*.

Furthermore, overexpression and repression of *OpWRKY3* affected the expression level of genes involved in biosynthesis of camptothecin, tryptamine, loganin, and secologanin in transgenic hairy roots. The *TDC* gene expression and loganin concentration were increased significantly while the *SLS* gene expression and secologanin concentration were decreased significantly in *OpWRKY3* overexpression hairy roots. This is consistent with a previous report that the accumulation levels of secologanin exhibited a strong negative correlation with the expression level of *TDC*, and that of loganin exhibited a negative correlation with the expression level of *SLS* [17]. These results suggested that *OpWRKY3* is involved in biosynthesis of tryptamine, loganin and secologanin, which as CPT biosynthetic precursors. Numerous reports showed that WRKY TFs regulate their target genes by binding to the W-box elements containing the TTGAC(C/T) motif [41]. Simultaneously, previous reports indicated that overexpression of *CrWRKY1-SRDX* repressed the transcription of *TDC* by binding to the W-box elements of its promoter [40]. *SmWRKY2* increased accumulation of tanshinones by binding to the W-box of the *SmCPS* [42]. The yeast one-hybrid experiments implied that *OpWRKY3* regulates the biosynthesis of camptothecin and its precursors by binding to the W-box elements of the *OpCPR* gene promoter. 

The total production (hairy roots and medium) of CPT has been increased significantly in OE-8, 13, 14 lines while it was significantly decreased in the S lines. However, the CPT concentrations in transgenic hairy roots were not significantly changed in OE and S lines, compared to control. It may be due to overexpression of *OpWRKY3* slightly increased the biomass of hairy roots while repression of *OpWRKY3* down-regulated the biomass of hairy roots. These results indicated that *OpWRKY3* influenced the production of CPT by regulating the growth and development of hairy roots. 

We found that *OpWRKY3-SRDX* decreased CPT production as quantified through a confocal microscope and high performance liquid chromatography (HPLC) method. The previous report has been reported that camptothecin was mainly accumulated in the root tip of *O. pumila* roots [43]. The camptothecin can emit blue auto-fluorescence under 360 nm UV light [43,44]. According to this feature, it was found that the camptothecin was mainly accumulated in glandular trichomes and secretory canals of the stem and leaf in *C. acuminate* [45,46]. Therefore, it requires direct and effective methods to further examine the concentration of camptothecin by confocal microscope observation. These results can accurately identify the content of camptothecin, which is helpful to explore the transport and accumulation of camptothecin in plants.

## 4. Materials and Methods

### 4.1. Plant Materials and Elicitor Treatments

The aseptic *O. pumila* plants were cultured as per the previously reported method [18]. Roots, stems, leaves, and petioles from *O. pumila* seedlings and hairy roots were gathered to detect *OpWRKY3* expression patterns. For the analysis of *OpWRKY3* expression patterns by different elicitors including 50 µM gibberellin (GA3), 200 µM methyl jasmonate (MJ), 50 µM acetylsalicylic acid (ASA), 50 µM salicylic acid (SA), and 100 µM abscisic acid (ABA) were used and water or ethanol was used as controls. Elicitor treatments were conducted on *O. pumila* hairy roots sub-cultured for 30 days infected with modified *A. tumefaciens* strain C58C1. The treated samples were harvested separately at different time points, including 0, 0.5, 1, 3, 6, 9, and 12 hour. All the treated materials were immediately frozen in liquid nitrogen and stored at −80 °C in a refrigerator.

### 4.2. The Gene Clone and Bioinformatics Analyses of OpWRKY3

Based on the sequence information of the WRKY transcription factor in *O. pumila* transcriptome databases, the full length of *OpWRKY3* was amplified using PCR. The amplified primers of the full-length coding sequence (CDS) of *OpWRKY3* are listed in Table S1. The physicochemical properties of the amino acids were determined with the ExPASy server (http://web.expas y.org/compu te_pi/). Homologous proteins were searched using the BLASTp program against a non-redundant (nr) protein sequence. Phylogenetic analysis of OpWRKY3 with the *Arabidopsis thaliana* and other species WRKYs was performed using the neighbor-joining method and 1000 bootstrap replicates in MEGA 7.0. Multiple sequences alignment analyses were performed between OpWRKY3 with six selected WRKYs from the same clade from the phylogenetic analysis using DNAMAN version 5.0.

### 4.3. Subcellular Localization of OpWRKY3

To investigate the subcellular localization of *OpWRKY3*, its full length of *OpWRKY3* was amplified and inserted into vector pMON530-GFP located at the restriction site of *Bgl* II and *Kpn* I to generate *pMON530-OpWRKY3-GFP*. The constructed expression vector was transferred into *Agrobacterium* strain ASE and injected into 60-day old tobacco leaves. GFP fluorescence was observed using the confocal microscope after 48 h of cultivation [47]. In total, 1 μg mL^−1^ 4, 6-diamidino-2-phenylindole (DAPI) was injected into tobacco leaves for 3 h before observation. A corresponding empty vector was considered as the control.

### 4.4. Construction of Plant Expression Vectors

To investigate the role *OpWRKY3* played in camptothecin biosynthesis, transgenic plants overexpress and repress vectors of the *OpWRKY3* gene were constructed. For the construction of the *OpWRKY3* overexpress vectors, the full length of *OpWRKY3* was cloned from the cDNA library and inserted into the modified vector pCAMBIA2300^sm^ located at the restriction site of *Spe* I and *BstE* II to generate pCAMBIA2300^sm^–OpWRKY3 (Figure S6) [33]. For the construction of the *OpWRKY3* repress vectors, the *OpWRKY3* gene was linked to an SRDX domain for gene expression repression based on the Chimeric REpressor gene-Silencing Technology (CRES-T) to make a chimeric gene (*OpWRKY3–SRDX*) construct, with the SRDX domain linked to the C-terminal end of *OpWRKY3* [48]. The *OpWRKY3–SRDX* was amplified by PCR with gene-specific primers pCAMBIA 2300^sm^-OpWRKY3-SRDX-KF-SpeI and 2300^sm^-OpWRKY3-SRDX-KR-BstEII and cloned into the pCAMBIA2300^sm^ vector (Table S1). A corresponding empty vector was considered as the control.

### 4.5. Acquisition of Transgenic Hairy Root Lines

Aseptic stems were separated from the *O. pumila* plants and cultured on B5 solid medium in the darkness for 2 days. The *A. tumefaciens* strain C58C1 containing the constructed vectors were used to infect the isolated stems. The transformation procedure was conducted according to a previous report [18]. The positive transgenic hairy roots were chosen for further study. For acquisition of positive transgenic lines, individual hairy root samples were collected for isolation of genomic DNA using the cetyltrimethylammonium ammonium bromide (CTAB) method [49]. Positive colonies were identified by PCR with the specific primers. All the primers used in this article are listed in Table S1. Fragments of the positive hairy roots were cut down and inoculated into 50 mL B5 liquid media and cultured for about 45 days under dark conditions with the rate of 120 rpm at 25 °C [18].

### 4.6. Quantitative Real-Time PCR

Total RNA was isolated from all collected samples by using the RNAprep pure Tissue Kit according to the instructions (TIANGEN, Beijing, China). Total RNA of 2 μg was used to perform the reverse transcription analysis using the RNaseH-Kit and Cloned Ribonuclease Inhibitor Kit (TransGen Biotech, Beijing, China). Real-time quantitative PCR analysis was carried out to detect the expression pattern of *OpWRKY3* and eight genes involved in the camptothecin biosynthesis pathway (Figure S3). The expression level was normalized to the internal reference gene *Opactin*. The amplification reaction using was performed in Applied Biosystem Step One with an optional 48-well plate (Applied Biosystem) by the relative quantitative analysis method (2^−ΔΔCt^) [50]. The amplification profile was 95 °C for 10 min, followed by 40 cycles of 95 °C for 15 s, 60 °C for 30 s, and 72 °C for 30 s. All the primers used in this article are listed in Table S1.

### 4.7. Confocal Microscope Analysis

In order to observe the distribution of camptothecin in hairy roots, transgenic hairy root tips from cultures in B5 solid medium in the darkness for 20 days were used for temporary slide specimens. CPT fluorescence in all of samples was observed and photographed under a confocal microscopy with 360 nm UV excitation [43]. A corresponding empty vector was considered as the control.

### 4.8. Camptothecin Content Measurement

Positive transgenic hairy roots cultured for 45 days were collected for the determination of camptothecin content and production. The hairy roots were dried in an oven to a constant weight and ground to a powder and weighted. An aliquot of 0.1 g powder was weighed for extraction of camptothecin according to a previous report [18]. In order to detect camptothecin excreted into the medium, medium was extracted by equal volumes of chloroform and methanol (4:1) three times after that organic layer was separated and evaporated the organic solvent. Residue was dissolved in 2 mL of methanol for HPLC analysis. Different concentration of camptothecin standard (100, 83.3, 66.7, 50, 33.3 and 16.7 μg/mL) was used to describe the standard curve (Figure S7). The detection parameters were the same as that described previously [18]. CPT production per flask = CPT content in hairy roots × hairy roots biomass per flask + CPT content in medium × medium volume per flask.

### 4.9. Extraction and Analysis of Tryptamine, Loganin and Secologanin

Extraction method of trypamine and loganin was the same as camptothecin, while the secologanin was extracted from hairy roots with distilled water. The HPLC parameters of trypamine were as follows: The mobile phase consisted of acetonitrile, methanol, distilled water, and glacial acetic acid with the ratio of 43:30:26:1. The detection wavelength was 254 nm. The HPLC parameters of loganin and secologanin were as follows: The mobile phase consisted of acetonitrile and distilled water at a ratio of 1:3 (*v*/*v*). The detection wavelength was 236 nm. The flow rate was 1.0 mL/min with an injection volume of 10 μL. The standard curves of tryptamine, logan and secologanin were described using different concentrations of its standards (Figure S7). The production of tryptamine and loganin were calculated according to the formula of CPT production. The secologanin in medium was undetectable, so secologanin production per flask = Secologanin content in hairy roots × hairy roots biomass per flask.

### 4.10. Yeast-One Hybrid Assay

Yeast one-hybrid assay was performed according to previous report [50]. The coding sequence of full-length *OpWRKY3* was inserted into the *pB42AD* vector. Meanwhile, the *OpCPR* promoter sequences (from −80 bp to −86 bp and -433 bp to -439 bp, relative to translation start site) were constructed into a *pLacZ* plasmid, respectively. The resulting recombinant plasmids were co-transformed into yeast strain EGY48a. The transformed reporter strains were cultivated on SD/-Ura/-Trp medium for 48 h, and tested on SD/-Ura/-Trp medium with 5-bromo-4-chloro-3-indolyl-b-D-galactopyranoside (X-gal) for 24 h. The empty vectors *pB42AD* and *pLacZ* were used as negative controls.

### 4.11. Statistical Analysis

All experiments, including identification of positive hairy roots lines, qRT-PCR, HPLC analysis of camptothecin, and its intermediate compound, were performed three times and the results are shown as the mean values of three independent repeats ± standard deviation. Statistical significance was determined by the one sample *t*-test and one-way analysis of variance using SPSS 11.5 software (SPSS).

## Supplementary Materials

The following are available online at https://www.mdpi.com/1422-0067/20/16/3996/s1, Figure S1: Transformation of *O. pumila* explants for hairy roots lines. Figure S2: Expression pattern of *OpWRKY3* in transgenic hairy roots. Figure S3: The fresh weight (FW) and dry weight (DW) of *OpWRKY3* transgenic hairy roots after shake-flask culture. Figure S4: The content of CPT and its precursors in *OpWRKY3* transgenic hairy roots by HPLC. Figure S5: Camptothecin biosynthetic pathway in *O. pumila*. Figure S6: Expression vectors construction for transformation. Figure S7: Analysis of Tryptamine, Loganin, Secologanin and CPT standard by HPLC. Table S1: Oligonucleotide primers used in this study.

## Abbreviations

*O. pumila*	*Ophiorrhiza pumila*
CPT	Camptothecin
TIAs	Terpene indole alkaloids
GA3	Gibberellin
MJ	Methyl jasmonate
ASA	Acetylsalicylic acid
SA	Salicylic acid
ABA	Abscisic acid
TSB	Tryptophan synthase beta
TDC	Tryptophan decarboxylase
G10H	Geraniol-10-hydroxylase
10-HGO	10-hydroxy-geraniol oxidoreductase
SLS	Secologanin synthase
CPR	NADPH:cytochrome P450 reductase
STR	Strictosidine synthase
SGD	Strictosidine beta-glucosidase
TF	Transcription factors
