# Supplementary material for: Transcription Factor OpWRKY3 Is Involved in the Development and Biosynthesis of Camptothecin and Its Precursors in Ophiorrhiza pumila Hairy Roots
Source: Int J Mol Sci. 2019 Aug 16;20(16):3996. doi: 10.3390/ijms20163996 (PMC6719216; doi:10.3390/ijms20163996)
Supplement: Supplementary file 1 [file ijms-20-03996-s001.pdf]

Supplementary material

Figure S1 Transformation of *O. pumila* explants for hairy roots lines. (A) Wild *O. pumila* seedlings; (B) Aseptic *O. pumila* seedlings; (C) Stem explants for transformation; (D) Hairy root derived from the wounding sites of stems; (E) Single-line hairy root cultured on plate; (F) Hairy root cultured in B5 liquid medium in flask

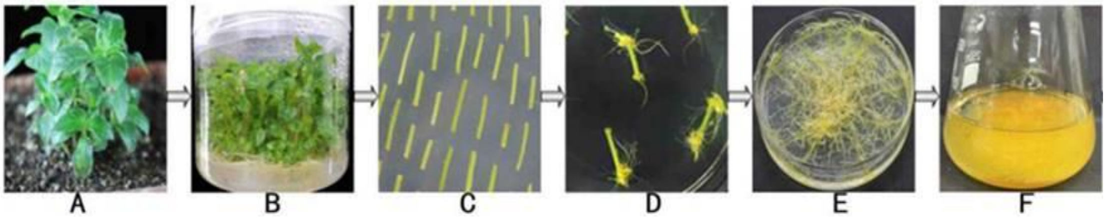

Figure S2 Expression pattern of *OpWRKY3* in transgenic hairy roots

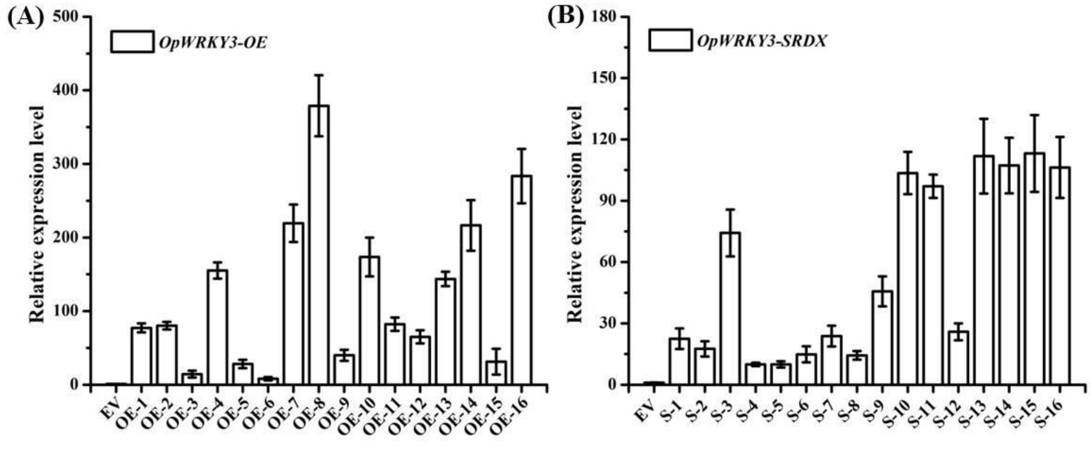

Figure S3 The fresh weight (FW) and dry weight (DW) of *OpWRKY3* transgenic hairy roots after shake-flask culture

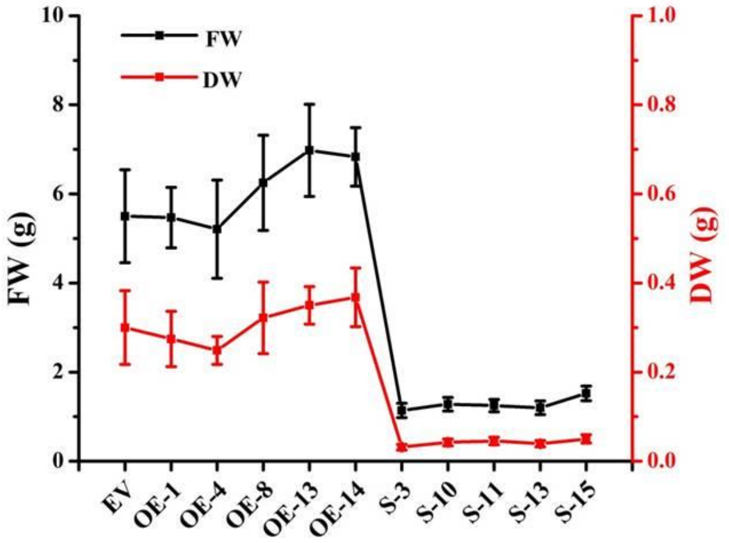

Figure S4 The content of CPT and its precursors in *OpWRKY3* transgenic hairy roots by HPLC. (A) The tryptamine content in *OpWRKY3* transgenic hairy roots by HPLC; (B) The loganin content in *OpWRKY3* transgenic hairy roots by HPLC; (C) The secologanin content in *OpWRKY3* transgenic hairy roots by HPLC; (D) The camptothecin content in *OpWRKY3* transgenic hairy roots by HPLC. Values are means ± standard deviation of triplicate analyses. ∗, significant at $P < 0.05$; ∗∗, highly significant at $P < 0.01$.

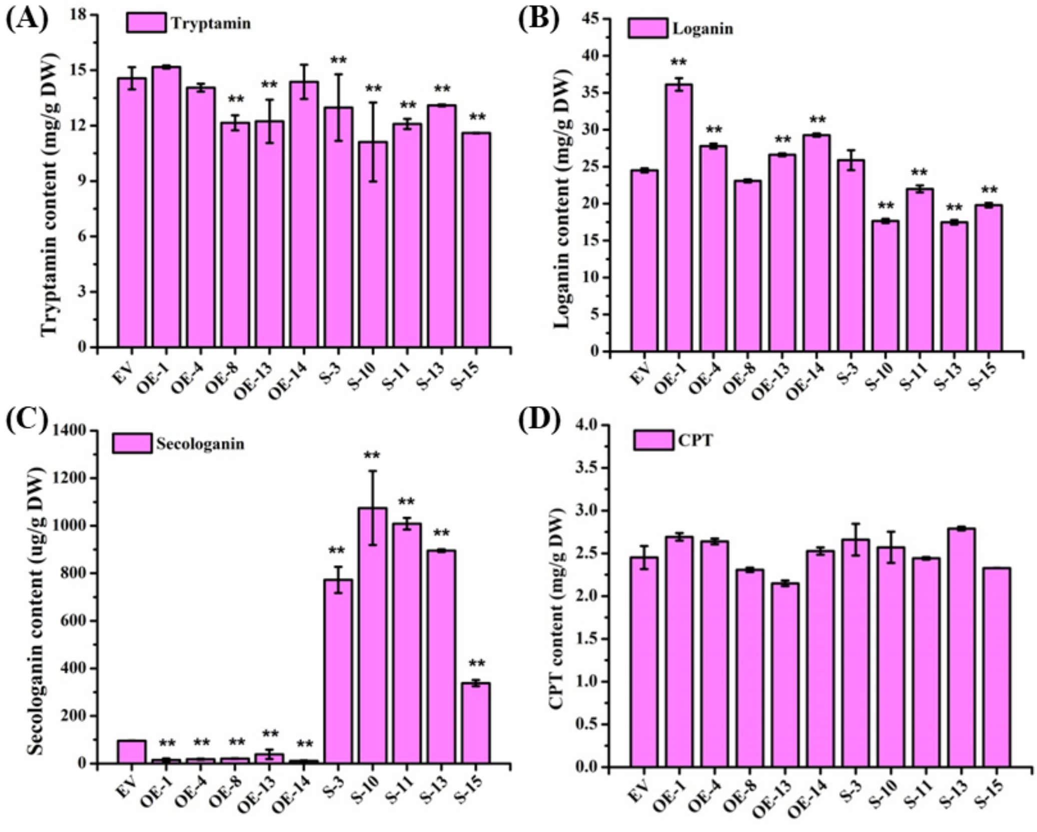

Figure S5 Camptothecin biosynthetic pathway in *O. pumila*. TSB, tryptophan synthase beta; TDC, tryptophan decarboxylase; G10H, geraniol-10-hydroxylase; 10-HGO, 10-hydroxy-geraniol oxidoreductase; SLS, secologanin synthase; CPR, NADPH-Cytochrome P450 reductase; STR, strictosidine synthase; SGD, strictosidine beta-glucosidase.

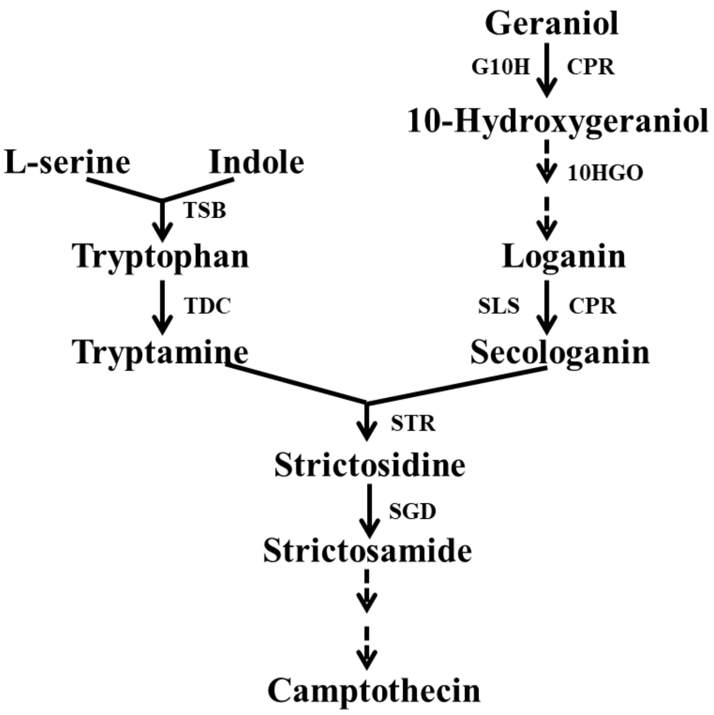

Figure S6 Expression vectors construction for transformation

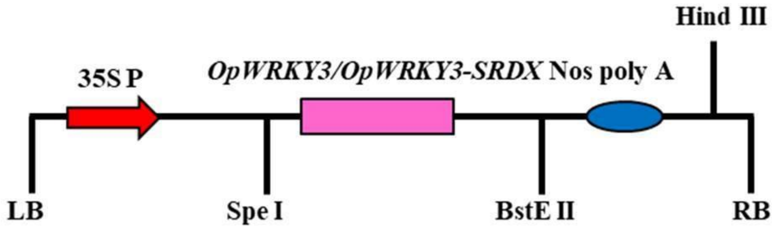

pCAMBIA2300[sm]::OpWRKY3/OpWRKY3-SRDX

Figure S7 Analysis of tryptamine, loganin, secologanin and CPT standard by HPLC

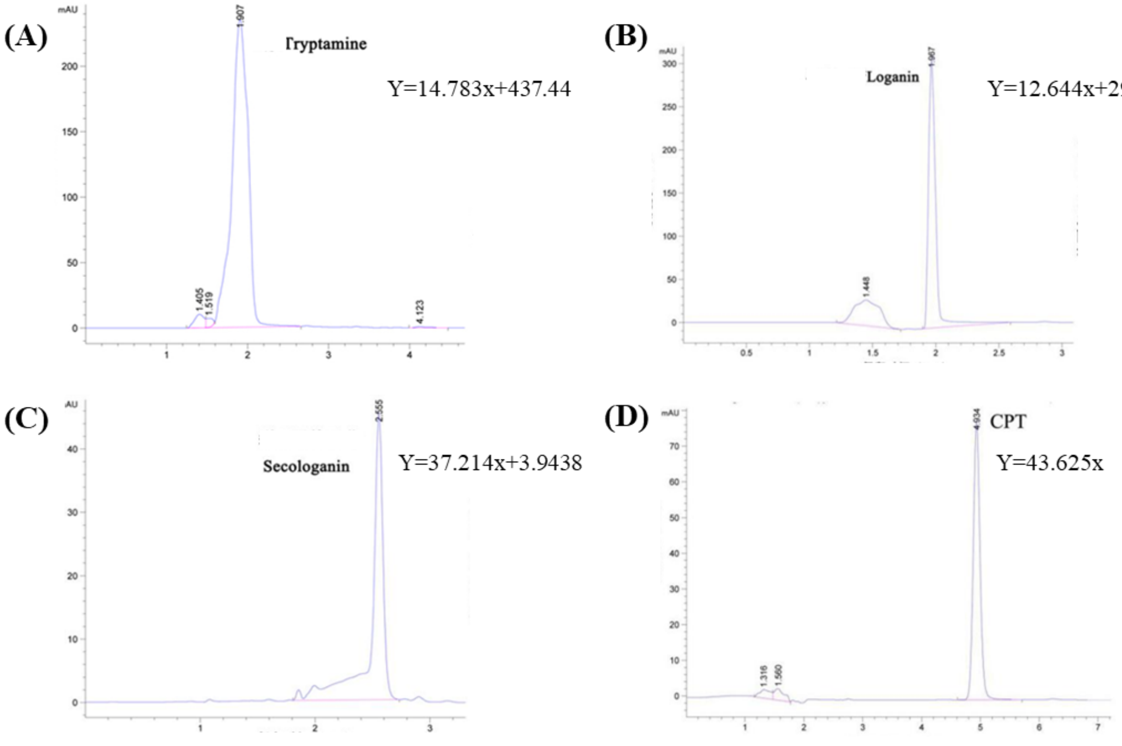

Table S1 Oligonucleotide primers used in this study

**Primer name**	**Primer sequence (5→3)**	**Application**
OpWRKY3-F1	CTTCAATTCTGCTCGTCTGTCGT	Cloning
OpWRKY3-R1	CAGGAGGTTTCCAGTTTCCACAA	Cloning
GFP-OpWRKY3-KF-BglII	GGAAGATCTATGGAGAATTCAGCAG	Subcellular localization
GFP-OpWRKY3-KR-KpnI	CGGGGTACCCCGGAAAAGAATCCTGA	Subcellular localization
2300^{sm}-OpWRKY3-OE-KF-SpeI	ACTAGTATGGAGAATTCAGCAG	Overexpression construct assembly
2300^{sm}-OpWRKY3-OE-KR-BstEII	GGTCACCTCAGGAAAAGAATCC	Overexpression construct assembly
2300^{sm}-OpWRKY3-SRDX-KF-SpeI	ACTAGTATGGAGAATTCAGCAG	Repression construct assembly
2300^{sm}-OpWRKY3-SRDX-KR-BstEII	GGTCACCTCAAGCAAAACCTAATCTAAGTTCCAGATCC AAATCCAAGGAAAAGAATCCTGAATTATC	Repression construct assembly
RolB-F	GCTCTTGCAGTGCTAGATTT	Positive identification
RolB-R	GAAGGTGCAAGCTACCTCTC	Positive identification
GusA-F	GTGAATCCGCACCTCTGG	Positive identification
GusA-R	ATCGCCGCTTTGGACATA	Positive identification
35S-F32	GAGGACCTAACAGAACTCGCC	Positive identification
OpWRKY3-R	CCCTCCCTAGTGTCAGCTCGTTTA	Positive identification
OpACTIN-QF	AGCAGCATGAAGATTAAGGTTGTG	qRT-PCR
OpACTIN-QR	CACATCTGCTGGAAAGTGCTG	qRT-PCR
OpWRKY3-QF	CCGACGCAACAAAATCCACA	qRT-PCR
OpWRKY3-QR	TGTTATCGGCCACAGATGGG	qRT-PCR
OpSTR-QF	AGCCATGGTTGTGTCGATTCT	qRT-PCR
OpSTR-QR	TTCACCATCGGAGTCAAAAGC	qRT-PCR
OpTDC-QF	TAGGCTCAATCCAGGGAAAGG	qRT-PCR
OpTDC-QR	TCCCACAGCAAACCTCAACA	qRT-PCR
OpCPR-QF	CCGGCAATATGAGCATTTCA	qRT-PCR
OpCPR-QR	GTTCTCGCCAAGCAGCAAA	qRT-PCR
OpG10H-QF	TGAGGAAGCCGATGTTTCCC	qRT-PCR
OpG10H-QR	TTTCATCACGCCCAATTGCC	qRT-PCR
Op10HGO-QF	TAATGGTTGGTGCACCGGAA	qRT-PCR
Op10HGO-QR	AGCTTTCAAGAGGCGTTCCA	qRT-PCR
OpSLS-QF	AAGCATCCCGAATGGCAAGA	qRT-PCR
OpSLS-QR	GAAGCATCACCGTTGGCATC	qRT-PCR
OpSGD-QF	ACCGGAAGGAGTGCCTATCT	qRT-PCR
OpSGD-QR	CCCCATTCCCATGTGTCCAA	qRT-PCR
OpTSB-QF	TGCAGGATGAAGATGGGCAG	qRT-PCR
OpTSB-QR	CAGAGTCGGGCACAGTTTCT	qRT-PCR
pB42AD-OpWRKY3-EcoR I-F	GATTATGCCTCTCCCGAATTCATGGAGAATTCAGCAGA TTGGGA	YIH
pB42AD-OpWRKY3-XhoI-R	TGGCGAAGAAGTCCACTCGAGTCAGGAAAAGAATCCT GAATTATCA	YIH
OpCPR-Pro EcoRI-W-boxII-F	aattcTTCTGGTCAAAACTTTCTGGTCAAAACTTTCTGGTC	YIH

	AAAACTc	
OpCPR-Pro XhoI-W-boxII-R	tcgagAGTTTTGACCAGAAAGTTTTGACCAGAAAGTTTTG<br>ACCAGAAg	YIH
